# Diagnostic Yield and Clinical Implications of Implantable Loop Recorders in Patients with Syncope in Germany: A National Database Analysis

**DOI:** 10.3390/jcm13061564

**Published:** 2024-03-08

**Authors:** Johanna Mueller-Leisse, Henrike Aenne Katrin Hillmann, Laura Iserloh, Bjoern Fruehauf, David Duncker

**Affiliations:** 1 Hannover Heart Rhythm Center, Department of Cardiology and Angiology, Hannover Medical School, 30625 Hannover, Germany; mueller-leisse.johanna@mh-hannover.de (J.M.-L.); hillmann.henrike@mh-hannover.de (H.A.K.H.); 2 Medtronic GmbH, 40670 Meerbusch, Germany; laura.c.iserloh@medtronic.com (L.I.); bjoern.fruehauf@medtronic.com (B.F.)

**Keywords:** implantable loop recorder, syncope, electrocardiogram, monitoring, atrial fibrillation, diagnostic yield

## Abstract

In patients with unexplained syncope, implantable loop recorders (ILR) are used to detect arrhythmias as a cause of syncope. This study aimed to assess the diagnostic yield and clinical implications arising from ILR implantation in patients with syncope in Germany. Secondary longitudinal data were obtained from a large German research database including anonymized data from nationwide statutory health insurances, representative for the German population. Patients ≥ 18 years with a diagnosis of syncope and ILR implantation between 2017 and 2018 were analyzed, and cardiac diagnoses and therapies during a follow-up period of two years assessed. Of 2,403,301 continuously insured persons in 2017–2018, 1360 (0.1%) received an ILR and 675 patients (45.6% female) were included. During follow-up, arrhythmias were diagnosed in 65.0%. The following antiarrhythmic therapies were established: pacemaker and defibrillator implantations in 20% and 1.5%, respectively, ablation therapy in 3.0%, and antiarrhythmic drug therapy in 4.7%. Aside from the diagnoses typically associated with syncope, atrial fibrillation or flutter was diagnosed in 37.0%, and anticoagulation therapy was initiated in 21.5%. There was a high diagnostic yield of arrhythmias following ILR implantation, leading to a relevant number of syncope-specific treatment. Arrhythmias not necessarily related to syncope were also diagnosed, leading to a high rate of anticoagulation therapy.

## 1. Introduction

Syncope is a common clinical issue with an approximate incidence of 6.2 per 1000 person-years and is associated with increased morbidity and mortality including sudden cardiac death for patients suffering from cardiac or unexplained syncope [1]. Establishment of a diagnosis is crucial for adequate treatment and prevention of future events [2,3,4,5]. However, the cause of syncope remains unknown in up to one third of patients [5,6]. As cardiac arrhythmias can be a cause for unexplained syncope, close rhythm monitoring is crucial. Holter monitoring and external loop recorders serve as main noninvasive monitoring strategies, but the diagnostic yield is low, especially in cases of infrequent symptoms [5,7]. Digital mobile or wearable devices are also increasingly used in the detection of arrhythmias [8,9,10]. However, recordings usually require patient activation at the time of symptoms. The use of such devices in the context of syncope is, therefore, limited. Moreover, reimbursement in daily clinical practice is lacking [11]. Next to noninvasive monitoring strategies, implantable loop recorders (ILR) are used to confirm the etiology of syncope [2,12,13,14,15,16,17,18], with a shorter time to diagnosis, more frequent treatment initiation, and a positive impact on survival compared to a conventional diagnostic workup [13,17,19]. These devices were first introduced in the 1990s [20] and have since been improved and miniaturized. They are inserted subcutaneously in the left pectoral region under local anesthesia, and they allow long-term continuous single-lead ECG monitoring. The availability of remote monitoring has further improved the diagnostic timing and follow-up with a potential economization of health care resources. The application of different ECG technologies in the workup of patients with syncope seems to be similar among Western countries [21]. While international guidelines recommend ILR implantation in patients with unexplained recurrent syncope after a standard workup [5,22,23,24], implementation in daily clinical practice is lagging behind, partly due to reimbursement issues [25,26,27,28,29,30]. In Germany, ILR implantation in the outpatients’ sector is usually associated with a high bureaucratic effort that includes writing individual reimbursement requests to health insurances attesting a proper diagnostic workup and indication according to guidelines [31]. Data on the diagnostic yield as well as clinical implications arising from ILR implantation following syncope in a real-world setting of the German population are scarce. The aim of this study was, therefore, to address this question, acquiring and analyzing national representative real-world data on current ILR implantations including follow-up data in Germany.

## 2. Materials and Methods

### 2.1. Study Design and Data Source

This is an observational study with a retrospective, longitudinal design using secondary data. The study was conducted following the guideline of “Good Practice Secondary Data” [32]. Data were obtained using the research database of the German Scientific Institute for Health Economics and Health System Research (WIG2). The WIG2 research database provides data representative of the German population with respect to age and gender distribution and includes anonymized data on the healthcare use and resource consumption of 4.5 million member records from nationwide statutory health insurances (SHIs) [33,34]. For analysis, SHI claims data of company health insurance funds (BKK) were used.

### 2.2. Patient Population

Patients ≥ 18 years with a diagnosis of syncope according to ICD-10-GM code R55 and an ILR implantation according to operation and procedure (OPS) code 5-377.8 between 2017 and 2018, who were continuously insured during a follow-up period of 24 months following the index day, were included in the analysis. The date of ILR implantation was referred to as the index day. 

To further refine the study population, the following exclusion criteria were applied: individuals with a primary diagnosis of stroke (ICD-10-GM 163) and/or ablation (OPS 8-835) at inclusion as well as patients with OPS-coding of ILR implantation (OPS 5-377.8), explantation (5-378.07), exchange (5-378.67), or revision (OPS, 5-378.57, 5-378.47, 5-378.87) and/or presence of a cardiac electronic device (ICD-10-GM Z95.0) within the year prior to the study initiation (2016). Patients who died during the follow-up period were included in the analysis and observed until their date of death.

Analyzed data included demographics, mortality, ILR-related cardiac diagnoses, mean time to first diagnosis, specific treatment initiation, and mean time to such treatment. Diagnoses were classified based on the International Statistical Classification of Diseases and Related Health Problems, 10th Revision, German Modification (ICD-10-GM). Procedures were classified based on the OPS codes. Medication is documented according to the Anatomical Therapeutic Chemical (ATC) classification system. All codes were checked for catalog changes.

ILR-related cardiac diagnoses analyzed included atrial fibrillation or flutter (I48), atrioventricular block or other cardiac conduction disease (I44, I45), and other cardiac arrhythmias including ventricular tachycardia, ventricular fibrillation, supraventricular tachycardia, and sick sinus syndrome (I49). Specific treatments analyzed included pacemaker implantations (OPS 5-377.1, 5-377.2, 5-377.3, 5-377.30, 5-377.31, 5-377.4, 5-377.40, 5-377.41, 5-377.k, 8-83d.3), defibrillator implantations (OPS 5-377.5, 5-377.50, 5-377.51, 5-377.6, 5-377.7, 5-377.70, 5-377.71, 5-377.j), ablation therapy (OPS 8-835), new treatment with anticoagulants (ATC B01 excluding B01AC, B01AD, and B01AY), and/or antiarrhythmic drugs (ATC C01 excluding C01E, C01D). 

### 2.3. Statistics

The statistical analysis was performed using SAS^®^ (SAS Institute Inc., Cary, NC, USA). The categorical variables are presented as numbers and percentages. The continuous data are presented as mean and standard deviation (SD).

## 3. Results

### 3.1. Study Population and Baseline Characteristics

**Figure 1:**
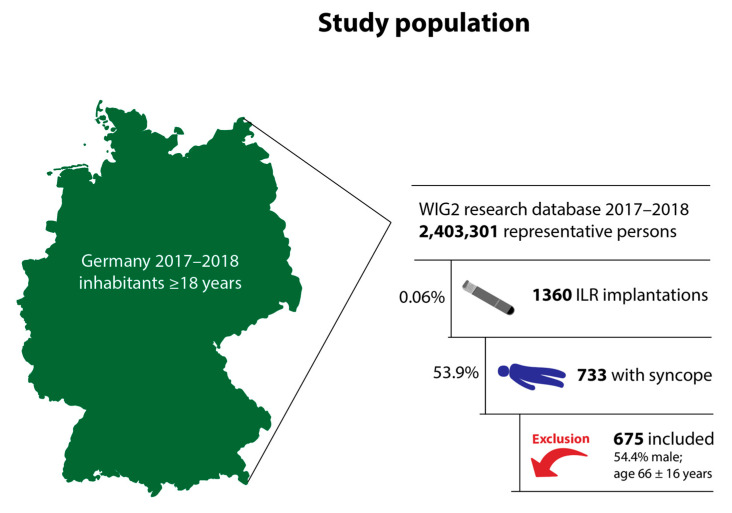
Study population. WIG2, research database of the German Scientific Institute for Health Economics and Health System Research; ILR, implantable loop recorder.

Of 2,403,301 continuously insured persons in the database in 2017–2018, 1360 (0.06%) received an ILR during the index period, and of these, 733 (53.9%) had a diagnosis of syncope. 58 patients met at least one of the exclusion criteria (prior cardiac device implantation code in 41 patients, ILR implantation or revision code in 8 patients, ablation code in 9 patients, and stroke in 7 patients). Thus, 675 patients were included in the analysis (Figure 1): 348 with implantation in 2017 and 327 patients with implantation in 2018. Projected to the German population, these numbers would correspond to 18,016 patients with syncope and ILR implantation in Germany within 2 years [34].

Of the included patients, 367 (54.4%) were male. Age at baseline was 66 ± 16 years (male patients 65 ± 16 years, female patients 68 ± 16 years; range 19–90 years). In total, 122 patients (18.1%) were on anticoagulants at baseline. A total of 20 patients (3.0%) were on antiarrhythmic drugs.

### 3.2. Follow-Up

During the two-year follow-up period, 47 patients (7.0%) died (26 patients within the first year and 21 patients within the second year). The most common primary inpatient diagnosis at their time of death was heart failure, in five patients (17.2%), followed by unspecific other diagnoses. 

Cardiac diagnoses during follow-up were established in 566 patients (83.9%), after 116 ± 162 days. In total, 439 patients (65.0%) received at least one of the predefined ILR-related diagnoses: An atrioventricular block or left bundle branch block was diagnosed in 59 patients (8.7%) after 206 ± 197 days, other cardiac conduction disease was diagnosed in 135 patients (20.0%) after 212 ± 213 days, and other arrhythmias were diagnosed in 275 patients (40.7%) after 176 ± 184 days. Atrial fibrillation or flutter was diagnosed in 250 patients (37.0%) after 212 ± 213 days. ILR-related cardiac diagnoses are presented in detail in Table 1.

In total, 159 patients (23.6%) underwent at least one predefined interventional therapy: 135 patients (20.0%) received a pacemaker, 10 patients (1.5%) were implanted with a defibrillator, and 20 patients (3.0%) received ablation therapy. A total of 167 patients (24.7%) were started on at least one predefined drug therapy: 145 patients (21.5%) were started on anticoagulation therapy and 32 patients (4.7%) were started on antiarrhythmic medication. The mean time to therapy was 225 ± 185 days for pacemaker therapy, 160 ± 136 days for defibrillator therapy, 338 ± 238 days for ablation therapy, 262 ± 228 days for anticoagulation therapy, and 227 ± 180 days for medical antiarrhythmic therapy, respectively (Table 2).

## 4. Discussion

This database analysis evaluated the diagnostic yield as well as the therapeutic consequences arising from current ILR implantations in patients with syncope in Germany using national representative real-world secondary data. The patient population was derived from a nationwide database representative of the German population. Most patients receiving an ILR within this population had a diagnosis of syncope (53.9%), which is in line with previous data from an international ILR registry [35].

The main findings of the present study are

1. Overall yield of cardiac arrhythmia diagnoses in patients with syncope and ILR was high at 65% during a follow-up period of two years.
2. Interventional antiarrhythmic therapies were established in 23.6%, including pacemaker implantations in 20.0%.
3. Aside from therapies for the prevention of syncope, new anticoagulation therapy was also initiated in 21.5%.

### 4.1. Diagnostic Yield after ILR Implantation

Following ILR implantation in patients with syncope, most patients (65%) were diagnosed with arrhythmias within two years of follow-up. These included diagnoses leading to pacing indication, such as conduction system disorders, but also atrial fibrillation or flutter, diagnosed in 37% of the population. Hence, the ILR provided a high diagnostic yield in terms of cardiac arrhythmias in patients with syncope, not restricted to arrhythmias directly related to syncope [12,13,14,15,17,19,35,36] but including other diagnoses with therapeutic implications such as atrial fibrillation. These findings are in line with other studies showing a high prevalence of atrial fibrillation in patients receiving an ILR. These studies not only included patients with cryptogenic stroke [37,38,39,40], but also patients with other indications such as syncope [19,35,41]. 

As the mean number of days to diagnosis in this analysis was more than 200 days, short-term monitoring using telemetric devices or Holter-monitoring in comparison to long-term monitoring using an ILR would not have been a sufficient diagnostic approach for the establishment of a diagnosis in these patients. 

### 4.2. Therapeutic Implications

A relevant number of patients in this analysis underwent pacemaker or defibrillator implantation within two years of follow-up (20.0% and 1.5%). Results of previous studies from Germany showed a similar [19] or even higher number in data from the PICTURE registry [13] of device implantations following ILR insertion after unexplained syncope. Again, most device implantations occurred only after several months following ILR insertion. Together with other data, this study emphasizes the role of long-term monitoring using the ILR in the establishment of causal therapies to prevent recurrent events in patients with syncope.

Next to device implantations as a therapy preventing syncope, a relevant number of patients in this cohort received other new treatments, mainly anticoagulation therapy (21.5%). A smaller number of patients received new antiarrhythmic treatment such as ablation therapy (3.0%) or antiarrhythmic medication (4.7%). Previous studies analyzing the use of an ILR after syncope have also reported small numbers of patients receiving treatments not directly related to syncope during follow-up [13,19]. Our representative real-world data show that this is an unexpectedly common finding and, therefore, of relevant clinical significance. While in many cases these treatments may not directly prevent recurrent syncope, they have the potential to prevent other adverse events such as ischemic strokes, cardiac remodeling, and heart failure, and may reduce morbidity and mortality [42]. However, the LOOP trial has shown that in a population at risk of stroke, an ILR resulted in an increased detection of atrial fibrillation and initiation of anticoagulant therapy without significantly reducing the incidence of ischemic stroke [43]. It remains unclear whether patients with a history of syncope and diagnosed atrial fibrillation via an ILR benefit from specific treatment. Randomized data should, therefore, be acquired from a larger population of patients diagnosed with atrial fibrillation after syncope, to investigate the morbidity and mortality benefit of putting them on certain treatments, especially oral anticoagulation.

### 4.3. Limitations

This study has several limitations. As the data were obtained using a research database and diagnoses/procedures were classified based on the ICD-10-GM/OPS codes which were required for billing purposes only, this may have led to over- and under-diagnoses of disorders. Moreover, ICD-10-GM codes only included grouped diagnoses not allowing for a detailed analysis. Formally, the ICD-10-GM code R55 includes syncope as well as collapse, which may have led to the inclusion of patients without actual syncope. However, as ILR reimbursement in Germany requires evidence of indication beyond ICD-coding, it is unlikely that a relevant number of patients without syncope were included. Patients with a history of stroke were excluded from the analysis. Another limitation of the study is that no detailed data on diagnostic workup before ILR implantation are available. Finally, data analyzed only include a two-year follow-up, and no further follow-up beyond pacemaker/defibrillator implantation, ablation therapy or initiation of oral anticoagulation or antiarrhythmic medication. Hence, other relevant findings may be missed.

## 5. Conclusions

Patients being implanted with an ILR for the indication of syncope in Germany show a high overall yield of cardiac diagnoses during follow-up. Diagnoses do not only include those that lead to syncope, but also others such as atrial fibrillation. Establishment of diagnoses lead to therapeutic consequences in many cases, including not only the implantation of a pacemaker or defibrillator, but also other treatments such as the beginning of anticoagulation therapy in a relevant number of patients. 

## Figures and Tables

**Table 1:** New cardiac diagnoses during follow-up. AV—atrioventricular.

Diagnosis	n (%)	Mean Time to Diagnosis (Days)
Any cardiac diagnosis	566 (83.9)	116 ± 162
AV block ^1^	59 (8.7)	206 ± 197
Cardiac conduction disease ^2^	135 (20)	212 ± 213
Other arrhythmias ^3^	275 (40.7)	176 ± 184
Atrial fibrillation ^4^	250 (37.0)	212 ± 213

^1^ I44, ^2^ I45, ^3^ I49, ^4^ I48: according to the International Statistical Classification of Diseases and Related Health Problems, 10th Revision, German Modification (ICD-10-GM).

**Table 2:** New anti-arrhythmic therapies during follow-up.

Therapy	n (%)	Mean Time to Diagnosis (Days)
Pacemaker	135 (20.0)	225 ± 185
Defibrillator	10 (1.5)	160 ± 136
Ablation	20 (3)	338 ± 238
Anticoagulation	145 (21.5)	160 ± 136
Antiarrhythmic therapy	32 (4.7)	227 ± 180

## Data Availability

Upon reasonable request, data are available from the corresponding author.
